# Varicocele and testicular cord torsion: immune testicular microenvironment imbalance

**DOI:** 10.3389/fcell.2023.1282579

**Published:** 2023-11-30

**Authors:** Vanesa A. Guazzone, Livia Lustig

**Affiliations:** ^1^ Universidad de Buenos Aires, Facultad de Medicina, Departamento de Biología Celular e Histología/Unidad Académica II, Buenos Aires, Argentina; ^2^ Consejo Nacional de Investigaciones Científicas y Técnicas (CONICET)—Universidad de Buenos Aires, Instituto de Investigaciones Biomédicas (INBIOMED), Buenos Aires, Argentina

**Keywords:** testis, varicocele, testicular cord torsion, autoimmunity, testicular inflammation, infertility

## Abstract

The main functions of the testis, steroidogenesis and spermatogenesis, depend on the endocrine axis and systemic and local tolerance mechanisms. Infectious or non-infectious diseases may disturb testicular immune regulation causing infertility. Literature has illustrated that bacterial and viral infections lead to autoimmune infertility: either sperm antibodies or autoimmune epidydimo-orchitis. However, little is known about the association between non-infectious testicular pathologic diseases and autoimmunity. Here we review the novel aspect of varicocele and testicular cord torsion pathology linked to inflammation and discuss how immune factors could contribute to or modulate autoimmunity in ipsi- and contralateral testis.

## Introduction

Infertility affects 10%–15% of couples, the male factor being present in around 50% of couples attempting to conceive. Moreover, global findings on sperm count strongly suggest a significant decline in male reproductive health, which seriously affects fertility (Turner et al., 2020).

The causes of male infertility or subfertility can be categorized mainly as genetic (autoimmune regulator (AIRE) gene deficiency), acquired, or idiopathic. Some acquired conditions are infections, testis tumors, environmental factors, hormonal factors, lifestyle, and systemic diseases. Numerous bacteria and viruses infect epididymis and testis through an ascending canalicular or haematogenous via, respectively. Hasan et al. (2022) describe these entities, mechanism of disease and negative impact on spermatogenesis and steroidogenesis. Microbial antigens may cross-react with testis antigen via molecular mimicry at the T- or B-cell level, and autoimmune orchitis may be the sequel of infections (Lustig et al., 2019).

Male infertility associated with immunological mechanisms may depend on the presence of: i) immune cells in the testis and excurrent ducts secreting cytokines and other inflammatory factors and/or ii) the presence of sperm antibodies. In the first case, immunopathological damage of the testis (and excurrent ducts) occurs through lymphomononuclear cell-mediated mechanisms triggered by antigens or pathogens that disrupt testicular immunoprivilege. In the second case, the antibodies generated may result in infertility by a variety of mechanisms (e.g., sperm agglutination, reduction of sperm motility and ability to penetrate the cervical mucus, inhibition of sperm capacitation, acrosome reaction and impairment of sperm-egg interaction).

A severe reduction in the male fertility potential is also observed in varicocele (VC) and to lesser degree in testicular cord torsion (TCT), two pathologies originating in embryological/anatomical defects. Despite extensive literature on VC and TCT, mechanisms involved in damage to the contralateral testis and in long-term negative effects on fertility are not yet clear. With this review, we propose a different approach to this topic through the analysis and relevance of inflammation and immunologic mechanisms found in these two pathologies and discuss how immune factors may contribute to or modulate autoimmunity in ipsi- and contralateral testis.

## Varicocele

VC is a vascular lesion manifested as abnormal dilatation and tortuosity of the pampiniform plexus veins, which occur by a pathological reflux of blood into the internal spermatic vein causing the elevation of scrotal temperature. It is most commonly observed on the left side (85% of cases), although some men are affected bilaterally. Isolated right-sided VC is rare. Reflux of blood is most often found on the left side as the hydrostatic pressure is higher on this side owing to the perpendicular drainage of the left internal spermatic vein into the left renal vein. VC can be observed in 35%–44% of men with primary infertility and 45%–81% with secondary infertility. Although VC repair (varicocelectomy) is a good way to improve a patient’s semen quality, postoperative natural pregnancy outcomes are still being debated (Jensen et al., 2017).

A comprehensive review of the pathogenesis of VC is found elsewhere (Cho et al., 2016). Current consensus is that pathophysiological mechanisms of VC -induced infertility are scrotal hyperthermia, hypoxia and oxidative stress. The hypoxia-inducible factor 1 (HIF), an intrinsic marker for tissue hypoxia, and nitric oxide (NO) are more highly expressed in the internal spermatic vein of patients with VC (compared to control patients) (Ozbek et al., 2000; Lee et al., 2006). Oxidative stress results from an imbalance of reactive oxygen species (ROS) and protective antioxidant system. ROS make functional contributions at appropriate concentrations (i.e., condensation of sperm nuclear chromatin during spermatogenesis), but quickly become destructive in excess. Heat stress and hypoxia produce a large amount of ROS, producing a negative impact on sperm quality and function via lipid peroxidation, mitochondrial dysfunction, DNA damage, and apoptosis (Wang et al., 2022).

However, some phenomena are not fully explained by the factors mentioned above; it is likely that other acquired risk factors and testicular immune microenvironment disorder contribute to irreversible testicular damage. Testicular biopsies in subfertile/infertile patients with left VC showed bilateral pathological alterations of seminiferous epithelium, sloughing of immature cells into the lumen of the tubules, and an increase in the interstitium tissue area (Dubin and Hotchkiss, 1969; Terquem and Dadoune, 1981; Abdelrahim et al., 1993). An elevated number of leukocytes (La Vignera et al., 2015; Mongioì et al., 2020) and levels of proinflammatory cytokines (IL6, IL1ß, TNFα and IL18) and chemokines (IL8 and CXCL5) are observed in the seminal plasma of infertile patients with VC (Moretti et al., 2009; Nazari et al., 2017; Zeinali et al., 2017). In experimental rat model, the progression of VC induces upregulation of IL1ß, IL1α, IL6 and INFγ in ipsilateral testis (Sahin et al., 2006; Habibi et al., 2015). Fang et al. (2021) summarized cytokines reported in VC of human and animal model.

Immune factors alter the normal function of the blood testis barrier (BTB) by changing the expression of cell junction adhesion molecule and increasing its permeability. BTB, also known as the Sertoli cell seminiferous epithelium barrier, is formed by cell junctions of adjacent Sertoli cells at the base of the seminiferous tubules. It is constituted of multiple cell junction types including tight junctions, basal ectoplasmic specializations, gap junctions, and desmosome-like junctions. Testicular biopsies of men with VC but unknown reproductive potential revealed intact functionality of BTB by intercellular tracer studies utilizing lanthanum nitrate (Cameron and Snydle, 1980). Nevertheless, an experimentally induced VC demonstrated significantly decreased claudin-11 and N-cadherin expression in ipsilateral testis versus sham rat testis (Oh et al., 2016; Pan et al., 2018). Evidence also suggests that VC is associated with anti-sperm antibodies (ASAs) as summarized in Table 1.

**TABLE 1:** Antispermatic antibodies (ASA) in Varicocele and Testicular Cord Torsion (TCT).

		Reference	Time after surgical procedure for ASA detection	Sample	% rats sperm ASA+/ total # rats	
Varicocele	Experimental data	Shook et al. (1988)	28-32 days	Serum	100% / 8	
		Reference	ASA evaluation (before or after surgery)	Sample	% patients ASA+/ total # patients	
	Clinical data	Witkin and Toth (1983)	Before	Seminal plasma	30% / 10	
		Golomb et al. (1986)	Before	Semen and seminal plasma	62.5% / 32	
		Oshinsky et al. (1993)	Before	Semen	17% / 29	
		Knudson et al. (1994)	BeforeAfter (6 months)	Seminal plasma	28% / 32	
					32% / 22	
		Solis et al. (2001)	Before	Seminal plasma	46.7% / 137	
		Bozhedomov et al. (2014)	Before	Seminal plasma	13% / 367	
		Reference	Surgical Procedure	Time after torsion for ASA detection	Sample	% rats sperm ASA+/ total # rats
Testicular Cord Torsion	Experiemental data	Rodriguez et al. (2006)	TCT	30 days	Serum	100% /12
		Reference	Surgical Procedure	Time after torsion for ASA evaluation	Sample	patients ASA+/ total # patients
	Clinical data	Mastrogiacomo et al. (1982)	Detorsion or orchidectomy	6 months to 2 years	Serum	23% / 13
				More than 2 years		33% / 12
		Zanchetta et al. (1984)	Not described	7 years	Serum	8.8 % anti-Leydig cells 4.4% anti-germ or Sertoli cells

## Testicular cord torsion

TCT is a urological emergency affecting 1:4000 males under 25 years old. Early diagnosis (about 6 h) and immediate surgery (detorsion and orchiopexy) are important to avoid irreversible ischemia-induced damage of the testis (Danner et al., 1982). Most authors consider that gonad viability following testis torsion lasts 24 h¸ and that irreversible testis damage (edema, interstitial hemorrhage, apoptosis, sloughing of the germinal epithelium, and finally necrosis) occurs after that time. Prolonged testicular ischemia leads to an infarcted testis that should be removed (orchidectomy). Even patients treated by detorsion might become infertile or subfertile in the future, exhibiting ASA production, decreased sperm motility, and reduced sperm counts as long-term effects. Endocrine profiles are within normal range although serum testosterone is significantly lower only after orchidectomy or testicular atrophy which occurs in 47% of patients following surgical detorsion (Aggarwal et al., 2022).

Experimental models in rats and mice demonstrated that ischemia-inducing testicular torsion followed by torsion repair and reperfusion induces high levels of ROS and correlates with an inflammatory response expressed by upregulation of proinflammatory cytokines TNFα and IL1ß. These cytokines plus NO are involved in neutrophil recruitment into the testicular interstitium (Turner et al., 1997; Lysiak et al., 2003). In contrast, E-selectin knockout mice and wild-type mice rendered neutropenic showed a significant decrease and a reduction of germ cell specific apoptosis (Lysiak et al., 2001). E-selectin is an endothelial adhesion molecule in charge of tethering which allows rolling of neutrophils to endothelial cells.

Alterations of testis histopathology and function of the contralateral testis in TCT have been demonstrated by several authors in experimental TCT models (guinea pig, rabbits, and rats) (Nagler and White, 1982; Cerasaro et al., 1984; Chakraborty et al., 1986; Pakyz et al., 1990; Vigueras and Reyes, 2004) whereas human data are more limited (Chakarborty et al., 1980; Laor et al., 1990).

Rodriguez et al., 2006, analyzing the contralateral testis of adult male rats subjected to unilateral spermatic cord torsion showed, 30 days after torsion, focal damage of seminiferous tubules associated with inflammation characterized by a significant increase in the number of resident and inflammatory macrophages, T lymphocytes, and mast cells localized in the testicular interstitium. Mast cells might indirectly trigger germ cell damage and fibrosis of the seminiferous tubule walls when tryptase increases microvascular permeability, stimulating inflammatory cell migration and releasing cytokines. TNFα content in testicular fluid of rats with TCT was significantly higher than in the sham group, suggesting it could be involved in apoptosis of TNFR1 positive germ cells. Serum sperm antibodies were detected in TCT rats and also in human subjects (Table 1).

## Discussion

Systemic tolerance and local immune privilege are partners for complete immune protection of testis. Systemic tolerance involving antigen-specific regulatory T cells (Tregs) is maintained in peripheral lymphoid organs by continuously egressing germ cell antigens via transcytosis in Sertoli cells (Lustig et al., 2019). Testicular immune privilege also involves multiple mechanisms such as a BTB, secretion of numerous immunosuppressive factors mainly by macrophages, Sertoli, peritubular, and Leydig cells, and the presence of Tregs. Autoimmunity against spermatogenic cells develops as a consequence of the breakdown of local immune tolerance. Activation of lymphomonocyte cells during immune cell reactions against auto-antigens may trigger release of chemokines, cytokines and ROS (Lustig et al., 2020). Autoimmune infertility is often clinically silent and might be caused by VC and TCT. The higher VC incidence in men with secondary infertility suggests that men with prior fertility may suffer VC-mediated secondary infertility, and the presence of VC may cause a progressive decline in fertility (Naughton et al., 2001). Increased levels of proinflammatory cytokines, leukocytes, and ASAs in seminal plasma indicate that testis/epididymis inflammation occurs in VC and TCT. Proinflammatory cytokines appear to be a natural component of seminal plasma but their production increases in response to chronic inflammations, thereby playing a detrimental role in spermatogenesis. Sperm immaturity is more frequent in VC patients vs. controls, suggesting that it is associated with a detachment of cells from the seminiferous epithelium, also strengthened by the frequent presence of spermatocytes and spermatids in these ejaculates (Moretti et al., 2009). Disorganization of seminiferous epithelium and the presence of immune cells in ipsi- and contralateral testis observed in VC and TCT resembles the lesion in men with autoimmune orchitis. Orchitis is characterized by seminiferous tubules with progressive loss of germ cells that are replaced by granulomatous inflammation consisting of T cells, macrophages, dendritic cells, and multinucleated giant cells. These findings mimic the changes in experimental models of orchitis that have been valuable in understanding the pathogenic mechanism of testicular damage (Lustig et al., 2020).

Hypoxia and oxidative stress in the ipsilateral testis in VC and TCT induce dysfunctional testis consistent with apoptotic germ cells observed in men and rat testis. As in autoimmune orchitis, a hypoxic and oxidative microenvironment also induces HIF-1α and NO, respectively. In long-term experimental VC, damage to seminiferous tubules is more severe, apoptosis of spermatogenic cells and expression of HIF-1α gradually increase whereas antiapoptotic proteins Bcl-2 decrease in ipsilateral testis (Zhu et al., 2017). HIF-1α regulates the expression of vascular endothelial growth factor (VEGF) that could be involved in many VC and TCT pathophysiological effects. In an experimental model of autoimmune orchitis we demonstrate that involvement of VEGFA-VEGFR is associated with a significantly increased percentage of interstitial testicular blood vessels, suggesting that VEGFA might be an early marker of testicular inflammation (Gualdoni et al., 2021). Chakraborty et al. (1985) noted a significant increase in the percentage of total blood vessels in VC patients with severely affected testes compared to controls. An oxidative microenvironment is generated in orchitic testis by high levels of NO produced mainly by both resident and infiltrating macrophages. NO reaches seminiferous tubules and induces basal germ cell apoptosis by activating the mitochondrial pathway (Jarazo-Dietrich et al., 2012; Ferreiro et al., 2019).

Cytokines are produced and secreted by immune cells and by testicular somatic cells in response to external stimuli. Increased levels of proinflammatory cytokines IL1, TNFα, IL6, IL18 may be responsible for disruption of BTB in VC. To our knowledge, no study has evaluated BTB in TCT. During testicular inflammation BTB integrity is impaired—denoted by increased permeability to tracers. Concomitantly, changes in expression of cell junction adhesion molecules were detected (Pérez et al., 2011; Pérez et al., 2012).

Testicular injury via initial actions at the BTB to elicit subsequent damage to germ-cell adhesion, thereby leading to germ-cell loss, reduced sperm count, and male infertility or subfertility. In this context, there is an intricate relationship between the male gonad and the immune system resulting in production of antibodies against meiotic and postmeiotic germ cells. The presence of anti-sperm antibodies and the increase in the number of testicular immune cells in VC and TCT show that a humoral and cellular immune response occurs simultaneously with the histopathological damage, thereby suggesting involvement of an immunological mechanism that eventually impairs the contralateral testicular function. In fact, Gurdal et al. (2008) reported a trend of increasing apoptosis in bilateral testis correlating with increasing duration of VC. Wang et al. (2010) considered that the positive correlation between left- and right-testis HIF-1α expression and the left- and right-sided apoptotic index of germ cells as confirmation that left-sided VC could cause similarly bilateral testicular damage.

In early publications on TCT, an immunologic mechanism was also suggested as a possible mechanism involved in contralateral testis damage (Chakraborty et al., 1980; Dondero et al., 1980; Mastrogiacomo et al., 1982). Ozkan et al. (2001) reported that serum inhibin B levels (marker of Sertoli cell function and state of spermatogenesis) decreases after unilateral TCT, reflecting contralateral testicular damage. Measurement of serum inhibin B levels is more effective than histopathological examination.

An early orchiectomy following TCT prevents the release of spermatic antigens, the formation of antibodies, and damage to the contralateral testis (Nagler and White, 1982). Anti-lymphocyte IgG and splenectomy or corticoids before detorsion also prevent damaging of the contralateral testis (Nagler and White, 1982; Pakyz et al., 1990; Mogilner et al., 2006). However, Jacobsen et al. (2020) suggest that contralateral testis damage results from multifactorial processes that also include pre-existing congenital testicular dysgenesis (a maldeveloped male urogenital tract) that may predispose to TCT (Laor et al., 1990; Osemlak et al., 2021) and/or contralateral hypoxia following ipsilateral torsion.

In this review, we emphasize that VC, TCT, and orchitis respond to common mechanisms of inflammation-related male infertility and theorize that prolonged VC and TCT negatively impact on contralateral testis through an autoimmune response. Consistent with this hypothesis, inhibition of inflammation can alleviate VC- and TCT-mediated pathogenesis (Table 2).

**TABLE 2:** Effects of anti-inflammatory drugs in Varicocele and Testicular Cord Torsion.

		Drug	Effect	Reference
Varicocele	Experimental rat model	Resveratrol	< mRNA level NLRP3 inflammosome, caspase-1, Bax	Hajipour et al. (2018)
	Clinical data	Ketotifen fumarate (mast cell blocker)	Improves semen parameters, chromatin integrity and pregnancy rates	Azadi et al. (2010)
Testicular Cord Torsion	Experimental rat model	Cyclosporine + Prednisone	Improves sperm parameters	Pakyz et al. (1990)
			Reduces contralateral testis damage	
		Polydeoxy-ribonucleotide	> VEGF, VEGFR1, eNOS	Minutoli et al. (2011)
			Reduces ipsilateral testis damage	
		Melatonin	< Testis inflammation	Parlaktas et al. (2014)
			*<* Lipid peroxidation and enzymes activities	
			Improves spermatogenesis in the ipsilateral testis	Mirhoseini et al. (2017)
		Ketotifen fumarate	< Testis inflammation	Moreno et al. (2020)
			< Mast cell number	
			< Fibrosis of ST walls	
			Reduces damage of contralateral testis	
		Vitamin D3	Targeting ADAM 17 prevents germ cell apoptosis in the ipsilateral testis and protect contralateral testis	Mohamed et al. (2021)

Other drugs with exclusively anti- oxidative stress effects have not been included.
